# The δ^15^N in *Orbicella faveolata* organic matter reveals anthropogenic impact by sewage inputs in a Mexican Caribbean coral reef lagoon

**DOI:** 10.1007/s11356-023-30476-x

**Published:** 2023-11-03

**Authors:** Serguei Damián Rico-Esenaro, José de Jesús Adolfo Tortolero-Langarica, Roberto Iglesias-Prieto, Juan P. Carricart-Ganivet

**Affiliations:** 1 Laboratorio de Esclerocronología de Corales Arrecifales, Unidad Académica de Sistemas Arrecifales, Instituto de Ciencias del Mar y Limnología, Universidad Nacional Autónoma de México, Prol. Av. Niños Héroes S/N, Domicilio conocido, Puerto Morelos, Q. Roo 77580 México; 2 Departamento El Hombre y su Ambiente, Universidad Autónoma Metropolitana Unidad Xochimilco, Calzada del Hueso 1100, Col. Villa Quietud, Coyoacán Cd. de México, 04960 México; 3 Tecnológico Nacional de México/IT Bahía de Banderas, Crucero a Punta de Mita S/N, Bahía de Banderas, 63734 Nayarit, México; 4 Department of Biology, The Pennsylvania State University, 208 Mueller Lab, University Park, PA 16802 USA

**Keywords:** Organic matter, Hermatypic coral, Caribbean, Anthropogenic impact, Nitrogen inputs

## Abstract

Coral-reef ecosystems provide essentials services to human societies, representing the most important source of income (e.g., tourism and artisanal fishing) for many coastal developing countries. In the Caribbean region, most touristic and coastal developments are in the vicinity of coral reefs where they may contribute to reef degradation. Here we evaluated the influence of sewage inputs in the coral reef lagoon of Puerto Morelos during a period of 40 years (1970–2012). Annual δ^15^N values were determined in the organic matter (OM) extracted from coral skeletons of *Orbicella faveolata*. Average protein content in the OM was 0.33 mg of protein g^−1^ CaCO_3_ (±0.10 SD) and a 0.03% of OM relative to the sample weight (*n* =100). The average of N g^−1^ CaCO_3_ was 0.002% (± 0.001 SD). The results showed an increase (*p *< 0.001) in δ^15^N over the time, positively correlated with population growth derived from touristic development. These findings emphasize the need to generate urban-planning remediation strategies that consider the impact on natural environments, reduce sewage pollution, and mitigate local stressors that threaten the status of coral-reef communities in the Caribbean region.

## Introduction

In the last century, sewage inputs in tropical coastal ecosystems have become an ever-increasing problem that compromise the future of coral reef communities (Risk et al. 2009; Erler et al. 2020). Nitrogen (N) derived from human activities promotes extensive nutrient enrichment in these ecosystems transforming them from oligotrophic to eutrophic environments (Canfield et al. 2010; Erler et al. 2018). These anthropogenic sources of nitrogen such as fertilizers and wastewater may negatively affect the optimal functioning of many physiological and ecological processes in the coral reef system (Kimes et al. 2010; Lema et al. 2012).

The skeletal structure of some scleractinian corals displays an annual pattern of alternating high- and low-density bands, which is revealed when they are X-rayed (Knutson et al. 1972; Carricart-Ganivet 2007; Carricart-Ganivet and Barnes 2007). This property allows for the reconstruction of past environmental conditions over extended periods of time (Barnes and Lough 1993, 1996; Lough and Cooper 2011). Consequently, coral skeletons can be used to assess environmental effects on coral reefs, including sewage pollution, temperature, and seawater chemistry. They provide the means to evaluate sewage pollution and seawater quality on various spatial-temporal scales, offering crucial information for detecting potential threats and aiding in the improvement of management and conservation strategies (Risk et al. 2009; Erler et al. 2020).

Hermatypic corals build reefs through the accumulation of calcium carbonate (CaCO_3_) precipitated in their skeletons. The coral skeletons consist of a two-phase structure comprising fiber-like crystals of aragonitic calcium carbonate closely associated with organic molecules (organic matter = OM) trapped during crystal growth (Cuif et al. 1999; Muscatine et al. 2005; Erler et al. 2020). The OM components consist of glycoproteins and proteins rich in amino acids, such as aspartate and glutamate, which are synthesized by calicoblastic cells and secreted into the skeleton during the coral calcification process (Allemand et al. 1998; Clode and Marshall 2003; Muscatine et al. 2005; Drake et al. 2013; Takeuchi et al. 2016). The OM content may also contain insights from endolithic algae, bacteria, and particulate material, accounting for less than 0.1% of the total weight of CaCO_3_, and can remain intact over long-time scales, maintaining stability within the coral skeleton (Constantz and Weiner 1988; Allemand et al. 2003; Ingalls et al. 2003; Marion et al. 2005; Erler et al. 2016). Treatment of coral skeletal samples with HCl allows for the isolation of OM, enabling independent analysis of the nitrogen isotopes (^15^N/^14^N) ratio contained within the skeleton structure (Cohen and McConnaughey 2003; Muscatine et al. 2005; Kolasinski et al. 2008). The δ^15^N signal recorded in coral skeletons may depend on various factors, including (1) atmospheric fixation and the discharge of DIN from terrestrial runoff, (2) nitrogen fixation resulting from nutrient depletion in the system, and (3) proportional changes in autotrophic and heterotrophic nutrition due to coral metabolism (Yamasaki et al. 2011). δ^15^N values are calculated relative to the quantity contained in atmospheric nitrogen in parts per thousand (‰) using the following expression:

$${\updelta}^{15}\textrm{N}=\left[\left({\textrm{R}}_{\textrm{sample}}-{\textrm{R}}_{\textrm{reference}}\right)-1\right]\times {10}^3$$

where R is the ^15^N/^14^N proportion, and reference standard is the atmospheric nitrogen (Heaton 1986; Peterson and Fy 1987; Sulzman 2007; Risk et al. 2009). In this context, it is possible to quantify terrestrial runoff and groundwater nutrient inputs and the main sources of DIN enriched with ^15^N/^14^N (Gove et al. 2016; Erler et al. 2016). The enrichment of nitrogen (N) in coastal ecosystems is due to changes that occur in the nitrogen cycle, such as ammonia volatilization, denitrification, and nitrification, which are associated with sewage contamination (Heikoop et al. 2000; Risk et al. 2009; Sherwood et al. 2010). The isotopic composition (d^15^N) of recently fixed N oscillated from −3 to 0‰, while the DIN from sewage discharges exhibits δ^15^N values between +6 and + 22‰ (Heaton 1986; Sherwood et al. 2010; Cooper et al. 2009; Baker et al. 2010; González-De Zayas et al. 2011; Erler et al. 2020).

The Mexican Caribbean coast (MCC) has a particular nutrient-load input, due the karstic geomorphology and permeable limestone that interconnect terrestrial infiltration to groundwater systems, and in turn into coastal lagoons and reef-lagoon systems (Sánchez et al. 2013). The constant sea-ward freshwater flow increases the sewage-derived nutrients (e.g., nitrogen) input, which has increased over the past decade associated with the development of the tourist industry (Baker et al. 2013; Sánchez et al. 2013). Yet, annual variability on the isotopic concentration of nitrogen has been reported for seagrass meadows in different MCC localities with a different anthropogenic development level (Carruthers et al. 2005; Mutchler et al. 2007; Baker et al. 2010; Sánchez et al. 2013; Camacho-Cruz et al. 2020). At this point, it is important to determinate a baseline of nitrogen inputs through historical long-term δ^15^N analyses and a continuous monitoring of DIN. This information may reveal the impacts of population growth and tourism development, while improving conservation strategies and management politics aimed to mitigate anthropic threats in coastal ecosystems (Risk et al. 2009; Camacho-Cruz et al. 2020). In this study we analyzed δ^15^N in coral cores of *Orbicella faveolata* from the Puerto Morelos National Marine Park (PMNMP) at the Mesoamerican Barrier Reef (Mexican Caribbean). We measure δ^15^N using 40 years of historical composite record (1970–2012) in the coral skeleton-bound organic matter and their relationship with the human population increase to understand the influence of anthropogenic sewage discharge in the coral reefs of the Caribbean Sea.

## Materials and methods

### Study area

**Fig. 1:**
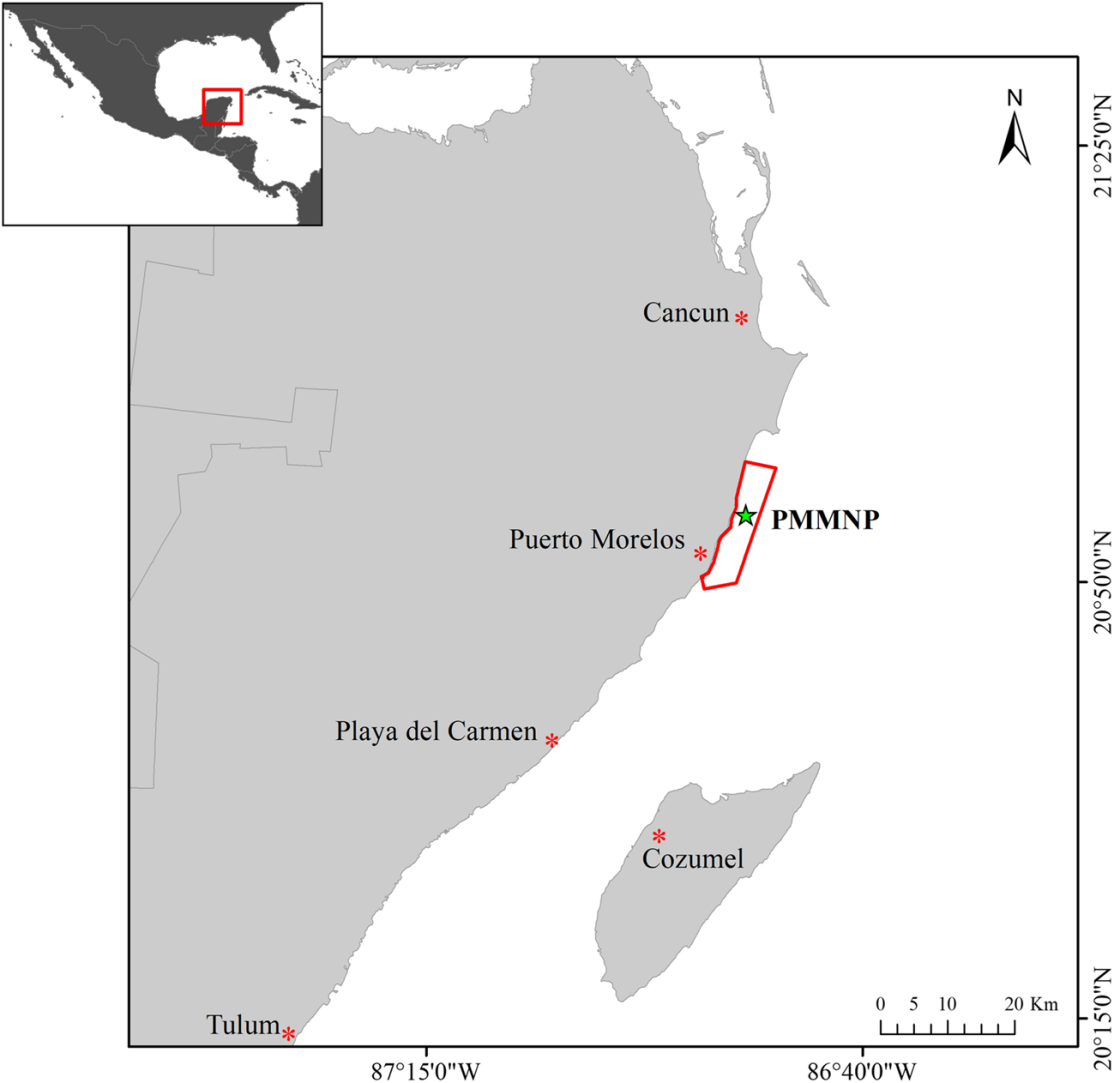
Collection site (green star) in Puerto Morelos Marine National Park, Mexico (PMMNP) at the Caribbean Sea. Red asterisks represent nearby important urban and tourism developments in the region

Puerto Morelos National Marine Park (PMNMP) is in the Riviera Maya between the cities of Cancun and Playa del Carmen; these locations represent the primary hubs of tourist development in the Mexican Caribbean. The basement of the Riviera Maya encompasses a karstic system with extensive aquifers that facilitate communication among them and discharge groundwater into the reef lagoon at a rate of 2,297,000 L day^−1^ per unit of surface area (Crook et al. 2011; Hernandéz-Terromnes et al. 2011; Iglesias-Prieto et al. 2014). The reef lagoon has an average depth of 3–4 m and ~500 m wide from intertidal zone and reef crest. The PMNMP has two connections to the open ocean: The Bocana reef at the north, which has a 300-m length with 6-m depth, and channel at the south, 400 m wide and 8-m depth. The primary inflow of water occurs above the reef crest, leading to a deeper water outflow through these two connections. In windy conditions, the current maintains the same direction in the surface layers. However, as depth increases, the current exhibits a different pattern (Coronado et al. 2007). Puerto Morelos lacks a well-structured system of sanitary or fluvial sewers (Vázquez-Lule and Adame 2009). The predominant infrastructure in the area comprises septic tanks that fail to meet the necessary sanitary standards (INE 2000). In the NMPPM there are at least thirteen submersed springs that discharge within the reef lagoon at 5-m depth (Pers. Obser.). This groundwater outputs ranges from 10-m-long “karstic fractures” to small circular depressions (seeps) only a few centimeters across (Crook et al. 2011). The flow of groundwater through the submarine springs is intermittent and ultimately controlled by sea level. During neap tides, submarine springs remain almost fully open, whereas during the spring tides, the springs close and open while tracking the semidiurnal tidal regime once the sea level rises above a threshold and the spring closes (Iglesias-Prieto et al. 2014) (Fig. 1).

**Fig. 2:**
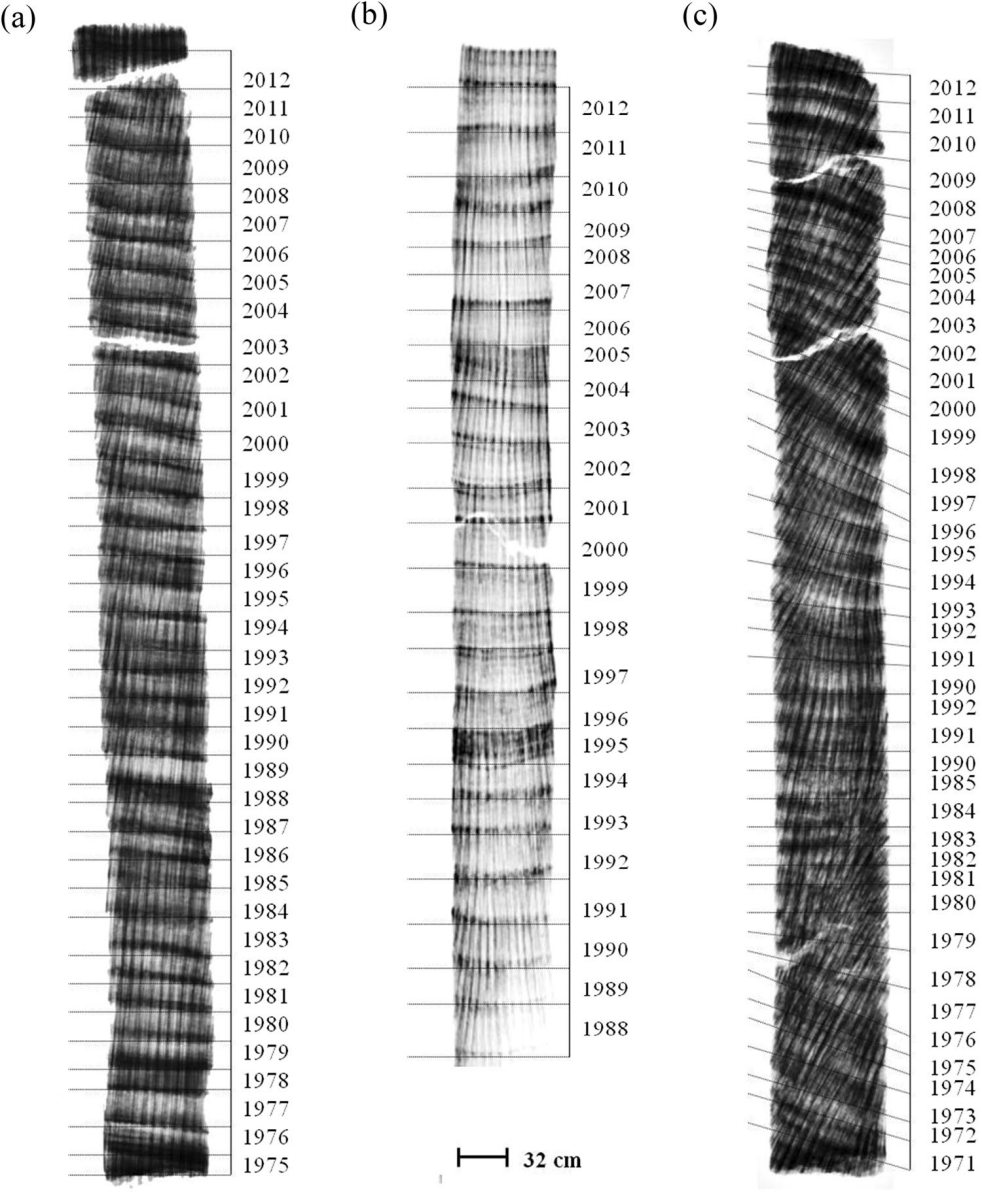
Positive X-ray images of *Orbicella faveolata* coral cores with annual dating (**a** from 1975 to 2012, **b** 1988 to 2012, and **c** 1971 to 2012) by the paired density bands pattern (high density= dark shades, and low density= light shades). Scale bar indicates coral cores length size

Coral cores of *Orbicella faveolata* colonies (*n*= 3) were obtained with a pneumatic drill at 5-m depth in La Bocana reef of PMNMP. The core samples were dried at 60°C using a conventional oven during 7 h. Then, coral cores were X-radiographed using General Electric X-ray machine (GE Hungay Rt. Medical Systems) with exposure settings at 73 kV for 20 mAs. The resulting digitalized images were dated retrospectively using the pattern of high- and low-density bands that represent an annual period (Fig. 2) (Lough and Cooper 2011). The OM extraction of annual skeletal fragments was done by wet chemistry; each high- and low-density band (two or three samples per year) was reduced to a fine coral powder and decalcified with HCl following the method proposed by Muscatine et al. (2005). The resulted solution was neutralized with NaOH 0.1 M and dialyzed with deionized water. The protein quantification was determined by aliquots of 3.0 ml per sample under the protocol proposed by Whitaker and Granum (1980). All samples were lyophilized, and annual OM extracts were taken to the Stable Isotope Biogeochemistry Laboratory of the Ohio State University to obtain the δ^15^N with a Costech analyzer coupled to a SIRMS Finnigan Delta IV Plus with continuous flow. Approximately 10% of all samples were run in duplicate. Stable nitrogen measurements (δ^15^N = ‰ deviation of ^15^N:^14^N relative to air) were made where the average standard deviation of repeated measurements of USGS41 standards was 0.09‰.

Historical record of population growth (habitants per year) was obtained from the National Institute of Statistics and Geography (INEGI, https://www.inegi.org.mx), and hotel occupancy and wastewater treatment data from the Secretary of Tourism of the Quintana Roo State (SECTUR, https://qroo.gob.mx/sedetur/) and the National Water Commission (CONAGUA, https://app.conagua.gob.mx/sistemasdeagua/) respectively. Statistical analyses were performed using R software (R Team core 2018).

## Results and discussion

Temporal changes of δ^15^N were reconstructed in *Orbicella faveolata* cores with an estimated age of ~40 years of coral growth (cores: A [1975 – 2012], B [1986 – 2012], and C [1971 – 2012], Fig. 2) and used as a sensitivity proxy for anthropogenic N inputs in Caribbean waters. Despite the statistical differences observed between the coral cores (*H* = 17.796, *p* < 0.001), these differences are likely attributed to variations in the number of years examined, with a maximum of 15 years. Average protein amount in OM per sample was 0.33 mg of protein g^−1^ CaCO3 (±0.1 SD, *n* =100) and a 0.03% of OM relative to the sample weight (Table 1). This result is lower than reported in other studies (0.8–1.5%, Cuif et al. 2004; 0.3%, Goffredo et al. 2011) 0.8–1% and 0.3% respectively, but closer to results reported by Constantz and Weiner (1988) who report OM values <​​ 0.1% of the sample weight. Differences can be attributed by the methods of extraction and quantification. The average of N g^−1^ CaCO_3_ was 0.002% (± 0.001 SD, *n*=106).

**Table 1:** Protein content and proportional organic matter (OM) estimation by core in *Orbicella faveolata*. Error reported as standard deviation

Core	Total protein (mg)	% OM	mg protein g^−1^ CaCO_3_
A	0.87 (±0.23)	0.03 (±0.1)	0.29 (±0.08)
B	0.94 (±0.29)	0.03 (±0.1)	0.31 (±0.10)
C	1.14 (±0.30)	0.04 (±0.1)	0.38 (±0.10)
Average	0.98 (±0.47)	0.03 (±0.02)	0.33 (±0.16)

**Fig. 3:**
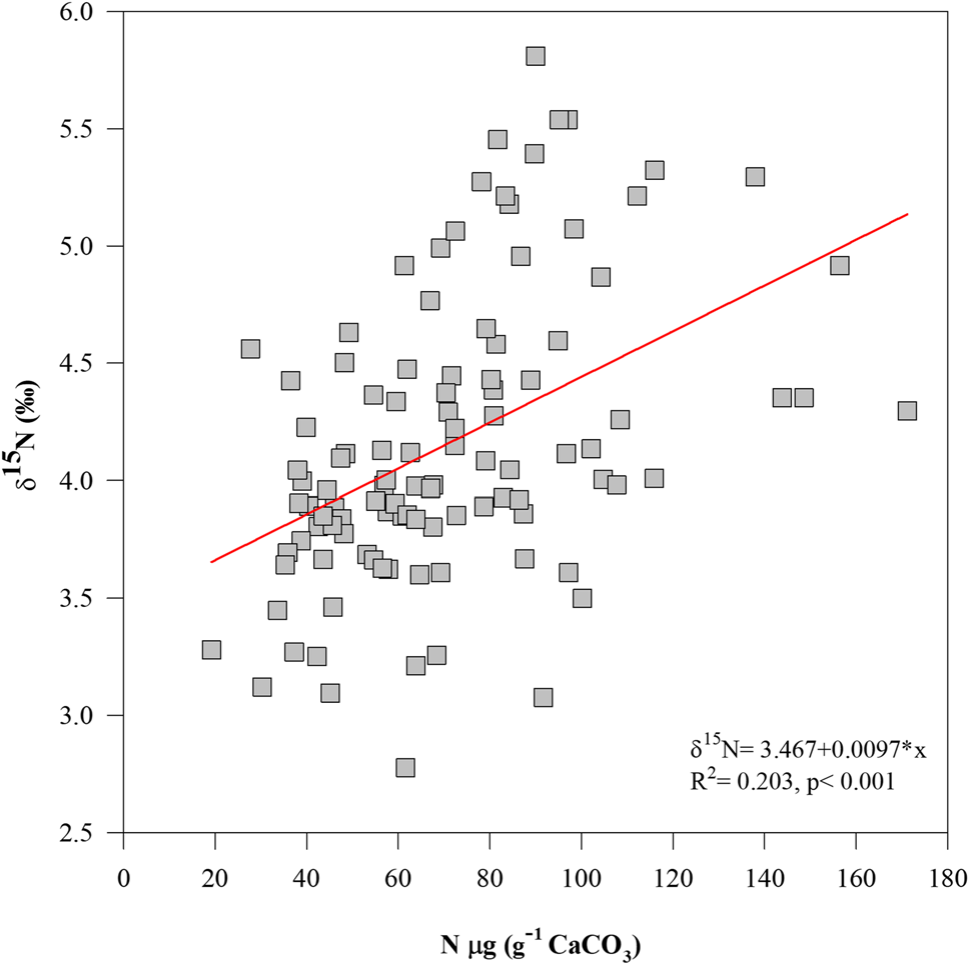
Scatterplots of mean annual δ^15^N vs. nitrogen (μg g^−1^ CaCO_3_) content in the organic matter of the coral *Orbicella faveolata*. A regression line and equation are also shown in the significant relationship (*p *< 0.001)

The mean δ^15^N values in the OM of *O. faveolata* were 4.16‰ which coincided with that reported by Muscatine et al. (2005) that described similar δ^15^N values (4.09 ±1.51‰) for many different coral species (*n*= 17). The nitrogen (μg) located in the coral OM of this study was positively related (*R*^2^= 0.203, *p *< 0.001) with δ^15^N (Fig. 3). The results showed a slight increase in OM production associated with the presence of heavier N isotopes in marine organisms as a coral. This OM increase is also reflected in several studies (Heikoop et al. 2000; Marion et al. 2005; Baker et al. 2010; Sherwood et al. 2010; Yamasaki et al. 2011; Baker et al. 2013), where the influence of various nitrogen sources, such as sewage discharges, fertilizers, water precipitation, and the residual contribution of upwelling, may impact the levels of δ^15^N in different coral reef organisms (Table 2).

**Table 2:** Values δ^15^N as N source proxies obtained from coral reef organisms in different localities

Source	Organism	Average δ^15^N ‰	Trend	δ^15^N ‰		Location	Reference
				Mín.	Max.		
Wastewater	Hermatypic coral	-	↑	+4.2	+9.3	Indopacific	Heikoop et al. (2000)
	Hermatypic coral	+8.4 ±2.2	↑	+4.2	+14.0	Sanur, Bali, Indonesia	Marion et al. (2005)
	Gorgonian	-	↑	+2.7	+4.7	Florida Reef Tract, Florida, E.U.	Ward-Paige et al. (2005)
	Seagrass	+9.1	↑	-	-	Cancún, Quintana Roo, Mexico	Carruthers et al. (2005)
	Gorgonian	+7.5 ±0.3	↑	+2	+10	FL, USA	Sherwood et al. (2010)*
	Gorgonian	+3.3	↑	+2.2	+4.3	Akumal, Quintana Roo, Mexico	Baker et al. (2010)
	Hermatypic coral	+4.2 ±0.6	↑	+2.8	+5.8	Puerto Morelos, Quintana Roo, Mexico	*This study*
Precipitation	Seagrass	+0.55	-	−1.83	+3.02	Cayo Coco, Cuba	González-De Zayas et al. (2011)
	Macroalgas	+2.67	-	+1.02	+4.17		
Fertilizers	Hermatypic coral	+7.3	↓	+15.1	+3.5	Lovina, Bali, Indonesia	Marion et al. (2005)
Fertilizers	Gorgonia	+3.8	↓	+1.3	+7.0	Antilles, North Atlantic and Yucatan, Caribbean	Baker et al. (2013)
Upwelling	Hermatypic coral	3.7	↑	+0.8	+8.3	Okinotori, Japan	Yamasaki et al. (2011)

**Fig. 4:**
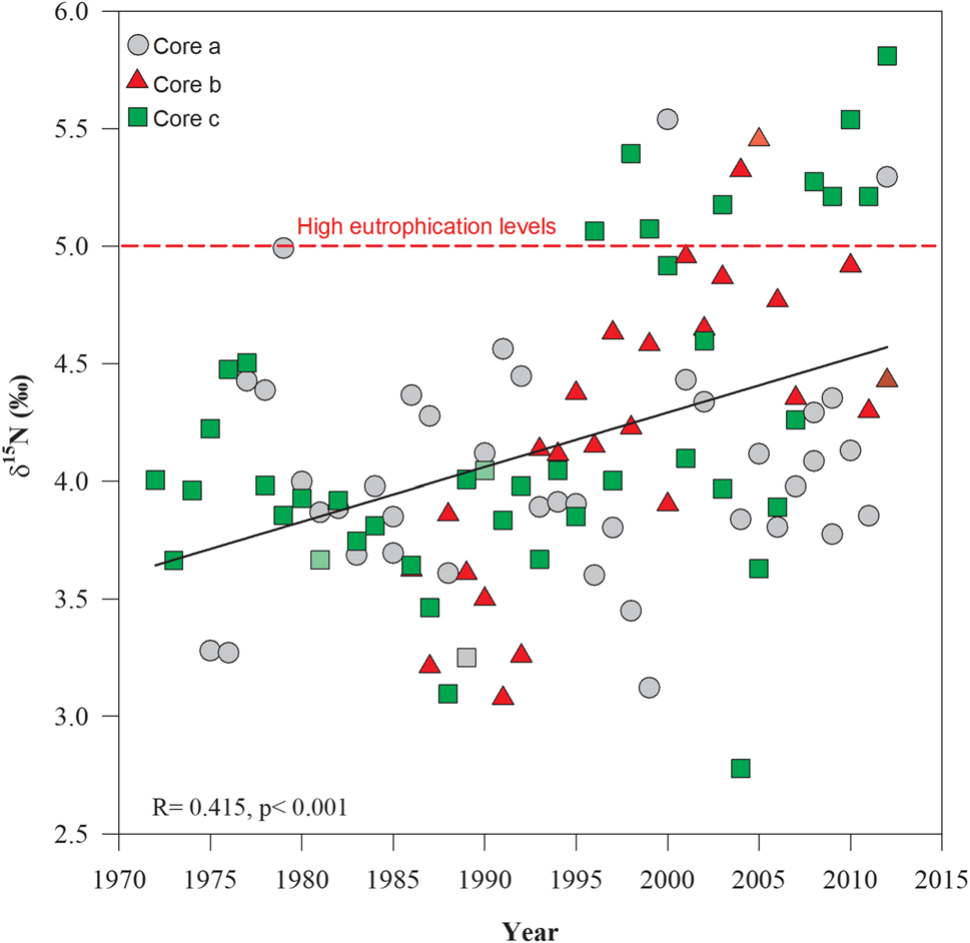
Temporal trend of mean annual δ15N in the organic matter of *Orbicella faveolata* cores (colored symbols). Red dashed line denotes values < 5.00‰

Annual δ^15^N values of all three coral cores presented in Figure 3 showed an increase over the time (1972–2012); this trend was statistically significant (*R*^2^= 0.172, *p < *0.01, Fig. 4). Maximum values (˃5.00‰) were presented between 1996 and 2012. Average δ^15^N values were presented between 1972 and 1992 (3.90 ± 0.40‰) with a significant increment between 1993 and 2012 of 4.40 ± 0.60‰ (Fig. 4). These data coincide with the population increase of Puerto Morelos from 1412 inhabitants (in 1990) to 9188 (in 2010), representing an increment of 600% in only a 20-year period. Hence, sewage related to the coastal development growth had an impact in the N inputs of the seawater in the reef lagoon rising the δ^15^N (>5.00‰); these values are closer to levels proper from sewage records (Fig. 4) (Cooper et al. 2009; Baker et al. 2010; González-De Zayas et al. 2011). This historical increase pattern is similar with the reported for other inshore coral reef nearby to human population development and high tourism fluxes (Baker et al. 2010; Erler et al. 2018). Under these conditions, large inputs of nutrients and contaminated water may promote ecosystem eutrophication and be related to declining water quality, the spread of enteric viruses, occurrences of coral diseases (such as white spots, white syndrome, and stony coral tissue loss), and the local extirpation of species (e.g., Acroporids) (Fabricius et al. 2005; Futch et al. 2010; Estrada-Saldívar et al. 2020; Cybulski et al. 2020).

**Fig. 5:**
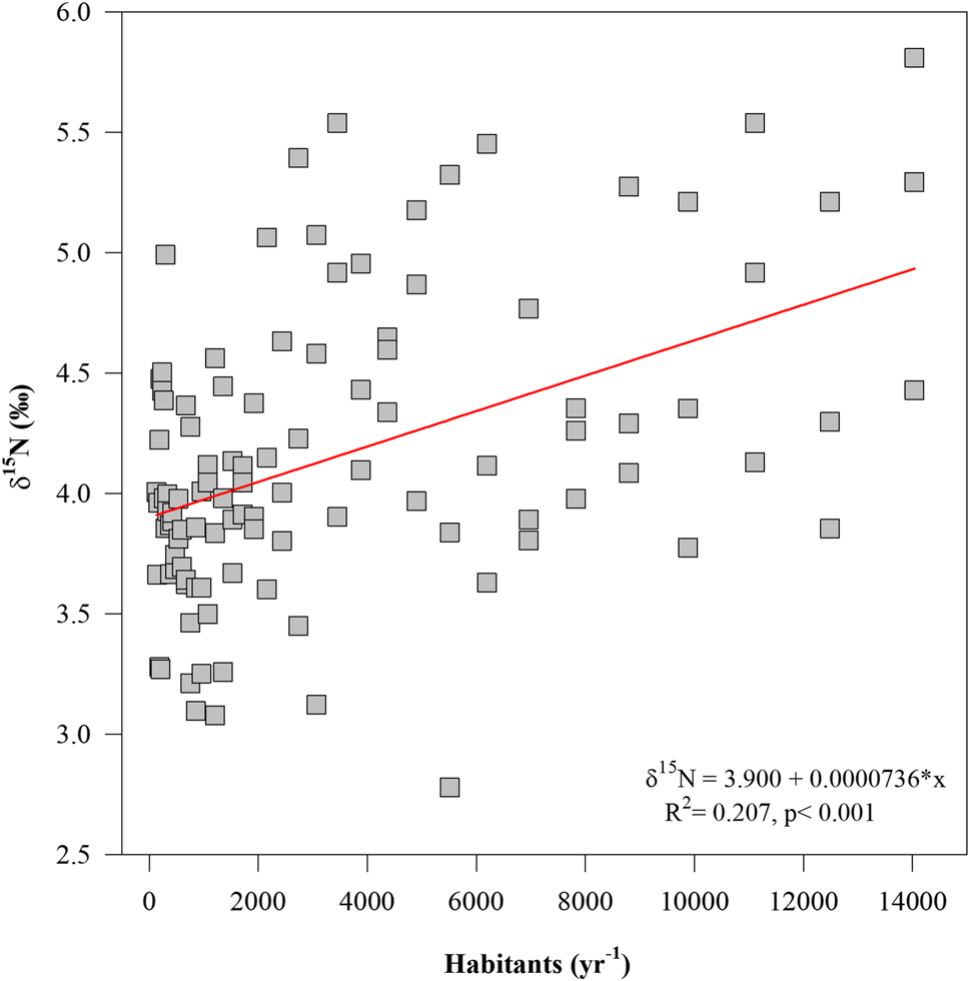
Positive relationship between number of inhabitants and δ^15^N annual values in *Orbicella faveolata* cores. A regression line and equation are shown

Annual values of δ^15^N were positive related with exponential human population growth (*R*^2^= 0.5348, *p <* 0.0001, Fig. 5). This observation suggests that contaminated water permeates into the aquifer representing an important contribution of N into the reef lagoon. This corroborates that anthropogenic activity may increase the N proportion in coastal waters, significantly shifting the δ^15^N in coral-reef ecosystems (Baker et al. 2010; Sherwood et al. 2010; Erler et al. 2020).

A positive annual trend of δ^15^N values with an approximated increase of ~0.55‰ per year^−1^ (0.10–1.48‰; Fig. 4) over >30 years from 1972 to 2012 resulted herein. Risk (2002) suggests that a 1.00‰ of δ^15^N is enough evidence of eutrophication processes to require mitigation strategies and the intervention of environmental policies. The high values of δ^15^N recorded in the last 40 years for this study may possibly lead to negative consequences, such as changes in nutrient cycles, which may promote shifts in community composition towards macro-algae dominance and the emergence of coral diseases (Alvarez-Filip et al. 2013; Lachs et al. 2019; Estrada-Saldívar et al. 2020; García-Sánchez et al. 2020; Cybulski et al. 2020; Contreras-Silva et al. 2020). Including the negative effect on the nitrogen and phosphorus cycle balance, ocasionally shifting states from oligotrophic to eutrophic states, which compromise the ecological functionality of coral reef ecosystems, occur (Fabricius et al. 2005; Paerl and Piehler 2008; Erler et al. 2020). Therefore, it is recommended to establish standards that include isotopic evaluations and annual monitoring protocols within the NMPPM and their nearby tourism development areas (Baker et al. 2010; Ladd and Collado-Vides 2013; Lachs et al. 2019).

The accelerated human population growth in the Mayan Riviera is the result of development policies oriented to massive touristic centers. Under this development scenario, the ecosystem needs to support not only the population growth derived from the people who work in this industry, but a floating population derived from hotel occupancy. In 2012 Puerto Morelos register a 72.5% average occupancy (Table 3); this means an annual average of 5516 visitors. During this year the average δ^15^N registered in OM was 5.20‰, the highest since 1972 (Fig. 4).

**Table 3:** Annual floating population from hotel occupancy in Puerto Morelos

Year	Hotel rooms	Average occupancy	Floating population
2008	2585	61.6	2389
2009	5036	53.8	4064
2010	5072	73.2	5569
2011	5072	74.3	5653
2012	5072	72.5	5516

**Fig. 6:**
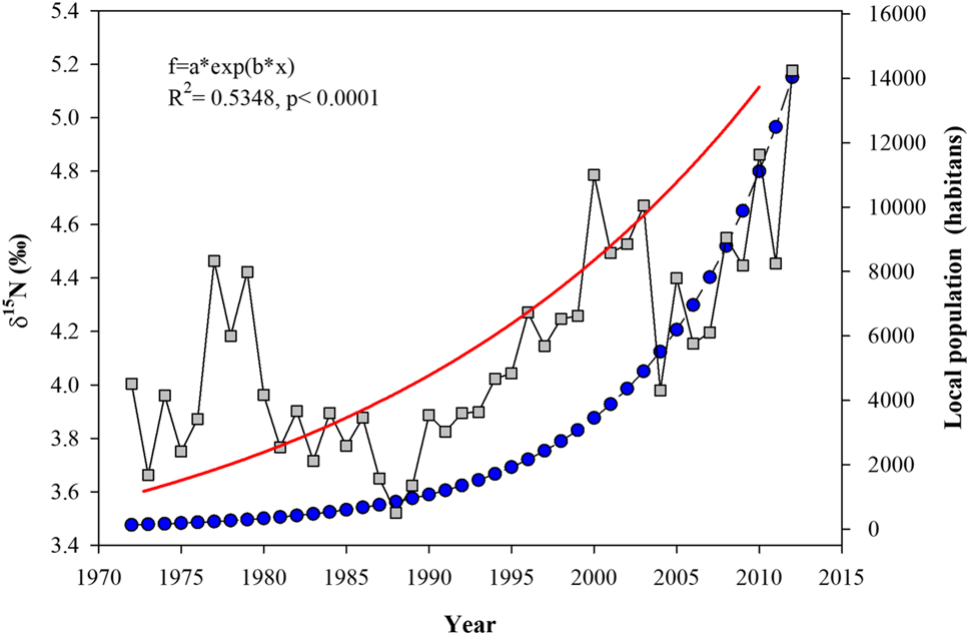
Mean annual δ15N in organic matter of *Orbicella faveolata* and population growth (total habitants) in Puerto Morelos from 1970 to 2010. Blue circles indicate number of habitants (based on national population census data). A regression (red line) and model equation are also shown for the relationship (*p *< 0.001)

According with the last National Inventory of Municipal Drinking Water Plants and Wastewater Treatment in Operation there are two treatment plants in Puerto Morelos. These are primary treatment plants based in activated sludge. The capability of this plants is 7 L s^−1^ and maintains a treaty rate of 2.5 L s^−1^; these plants discharge directly in the aquifer and flows into the reef lagoon (CONAGUA 2009). In 2010 Puerto Morelos recorded 9188 citizens had received an approximate of 2,297,000 L day^−1^ of water; according to the capability of this treatment plants only 52.7% was treated (Hernandéz-Terromnes et al. 2011). This means that approximately 1,087,400 L/day of untreated sewage were deposited into the groundwater of the aquifer in that year. Hernandéz-Terromnes et al. (2011) estimated nitrogen fluxes from untreated discharges in the karstic system and direct discharges into the reef lagoon, resulting in a nitrogen flux of 2.40-ton N km^−1^ year^−1^ (Fig. 6).

## Conclusion

This study reveals high levels of nitrogen in NMPPM’s coral reef ecosystem, indicating that actual sewage filtrations exceed the maximum parameters described by the Mexican policy and official standard for wastewater (Mexican Official Norm: 001-SEMARNAT-1996). Thus, the formulation of localized complementary environmental policies and mitigation strategies, tailored to the hydrologic region in the Yucatán Peninsula, becomes imperative. The establishment of marine protected areas has emerged as the primary conservation strategy aimed at preserving the integrity of coral reef ecosystems. However, the efficacy of this approach remains incomplete without the comprehensive inclusion of vital components, such as a well-structured wastewater system, within urban design frameworks. Furthermore, ensuring the preservation of interconnected habitats, including mangroves and groundwater systems, which demonstrate functional linkages, assumes significant importance (Michener and Kaufman 2007). Therefore, the implementation of environmental policies and the adoption of innovative urban development models represent the utmost priority in effectively addressing the detrimental impact of anthropogenic activities on coastal ecosystems in the Caribbean region.

## Data Availability

All data generated or analyzed during this study are included in this published article.
